# Multi-Layer and Conformally Integrated Structurally Embedded Vascular Antenna (SEVA) Arrays

**DOI:** 10.3390/s21051764

**Published:** 2021-03-04

**Authors:** Amrita Bal, Jeffery W. Baur, Darren J. Hartl, Geoffrey J. Frank, Thao Gibson, Hong Pan, Gregory H. Huff

**Affiliations:** 1 Department of Electrical and Computer Engineering, Texas A&M University, College Station, TX 77843, USA; abal@tamu.edu (A.B.); hongpan0507@gmail.com (H.P.); 2 Materials and Manufacturing Directorate, U.S. Air Force Research Laboratory, WBAFB, Dayton, OH 45433, USA; jeffery.baur@us.af.mil (J.W.B.); geoffrey.frank.ctr@us.af.mil (G.J.F.); 3 Department of Aerospace Engineering, Texas A&M University, College Station, TX 77843, USA; darren.hartl@tamu.edu; 4 Department of Material Science and Engineering, Texas A&M University, College Station, TX 77840, USA; 5 University of Dayton Research Institute, 300 College Park, Dayton, OH 45469, USA; thao.gibson@us.af.mil; 6 Department of Electrical Engineering, Pennsylvania State University, State College, PA 16801, USA

**Keywords:** unmanned aerial vehicle, phased array, frequency reconfiguration, beam steering

## Abstract

This work presents the design and fabrication of two multi-element structurally embedded vascular antennas (SEVAs). These are achieved through advances in additively manufactured sacrificial materials and demonstrate the ability to embed vascular microchannels in both planar and complex-curved epoxy-filled quartz fiber structural composite panels. Frequency-reconfigurable antennas are formed by these structures through the pressure-driven transport of liquid metal through the embedded microchannels. The planar multi-layer topology examines the ability to fabricate two co-located radiating structures separated by a single ply of quartz fabric within the composite layup. The multi-element linear array topology composed of microchannels embedded on to a single-layer are used to demonstrate the ability to conformally-integrate these channels into a complex curved surface that mimics an array of antennas on the leading edge of an Unmanned Aerial Vehicle (UAV). A parallel-strip antipodal dipole feed structure provides excitation and serves as the interface for fluid displacement within the microchannels to facilitate reconfiguration. The nominal design of the SEVAs achieve over a decade of frequency reconfiguration with respect to the fundamental dipole mode of the antenna. Experimental and predicted results demonstrate the operation for canonical states of the antennas. Additional results for the array topology demonstrate beam steering and contiguous operation of interconnected elements in the multi-element structure.

## 1. Introduction

Unmanned Aerial Vehicles (UAV) are deployed to serve a wide variety of commercial, municipal, and defense applications including health care, public safety, mobile network coverage diagnostics, etc. [1,2,3]. The growing utilization of UAV-based application spaces and the growing complexity of their missions have increased the need for more robust communication and sensing systems that support these wireless links. This has facilitated a need for robust, shape-conformal, and frequency reconfigurable antennas and phased arrays that can adapt to dynamic operational scenarios; these vehicles (controlled remotely or autonomously) are also required in many scenarios to maintain reliable and long-range communication with a ground station. Reconfigurable, multi-band, and/or broadband omnidirectional antennas used in UAV applications have been proposed in response to this (e.g., [4,5,6,7,8,9]). Antenna arrays have also been used in-place of single antennas in these applications to provide enhanced gain and scanning capabilities (e.g., [10,11,12]).

Antennas considered for use in UAVs are often derived from canonical planar topologies. The integration of these antennas on/in to the UAV presented challenges and numerous efforts have been carried out to embed these antennas into load bearing structures (even conformal to their surfaces). These efforts mainly focused on improving structural efficiency and antenna performance. Various efforts in this direction resulted in development of vertically grown carbon nanotubes and carbon composite fibers used for fabrication of load bearing antennas having desirable RF and mechanical performance [13,14]. Similar load bearing antennas, like Conformal Load bearing Antenna Structures (CLAS) and Composite Smart Structures (CSS), have been proposed to overcome geometrical limitations and eases integration with a non-planar surface [15,16,17,18,19]. Examples of CLAS include MEMS reconfigurable pixelated patch antennas and bi-layer log periodic antenna arrays [20,21]. Antennas and antenna arrays designed using high strength metal coated fibers suited for stress, weight and shape critical applications provide synergistic advances [22,23,24].

The development of load bearing antenna technologies has been critical in the deployment of radiating systems that can withstand the extreme environments and physical conditions encountered by both traditional aircraft and UAVs [25,26,27,28]. Advances in additive manufacturing using liquid metal as a reconfiguration mechanism [29,30] have been demonstrated recently to impart a greater degree of electromagnetic agility into the design space of conformal load bearing antenna systems. Previous work on the load-bearing, structurally embedded vascular antenna (SEVA) [30] demonstrated the frequency reconfigurability of a dipole antenna meandered in exponentially increasing sinusoidal pattern. The effective (resonant) length of the embedded dipole was varied using pressure driven transport of liquid metal (eGaIn) [31] to operate the antenna over a wide frequency range. The prior work [32,33,34,35] on embedded structures emphasize on the mechanics, materials, and fundamental concepts used to emphasize the functionalization of antennas in structurally embedded environments. The repeatability and stability of antenna characteristics make this structure an extensible demonstration vehicle to use in future and ongoing efforts. In this work the design is used to demonstrate the ability to engineer multi-layer and conformally curved reconfigurable antennas enabled by transport of liquid metal through the embedded microvascular channels. This is illustrated through two unique designs. First, it is used to demonstrate an antenna with vascular networks fabricated on two closely spaced and consecutive layer of fabric in the composite lay-up. In the second design it is shown that vascular networks with distinct pattern can fabricated in a complexly curved substrate mimicking the shape of a UAV wing. These structurally embedded vascular antennas operate over a decade of frequency in a single footprint. The organization of this work on multi-element frequency reconfigurable SEVAs follows. Full-wave electromagnetic solver [36] is used for simulation of these structures. First the design and fabrication of a multi-layer SEVA (ML-SEVA) is presented. The multi-element SEVA conformal linear array (SEVA-CLA) integrated onto a complex-curved surface is discussed next. The last section includes measured and simulated results from these structures. A brief discussion concludes the work.

## 2. Design and Fabrication

### 2.1. Multi-Layer SEVA (ML-SEVA)

**Figure 1:**
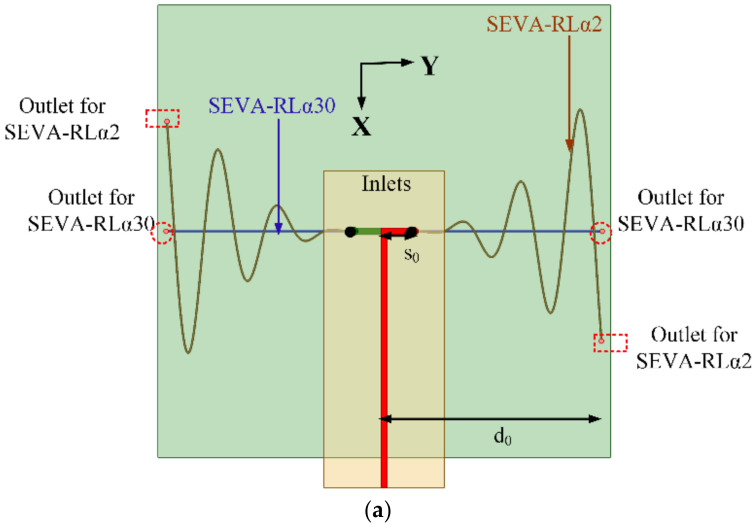

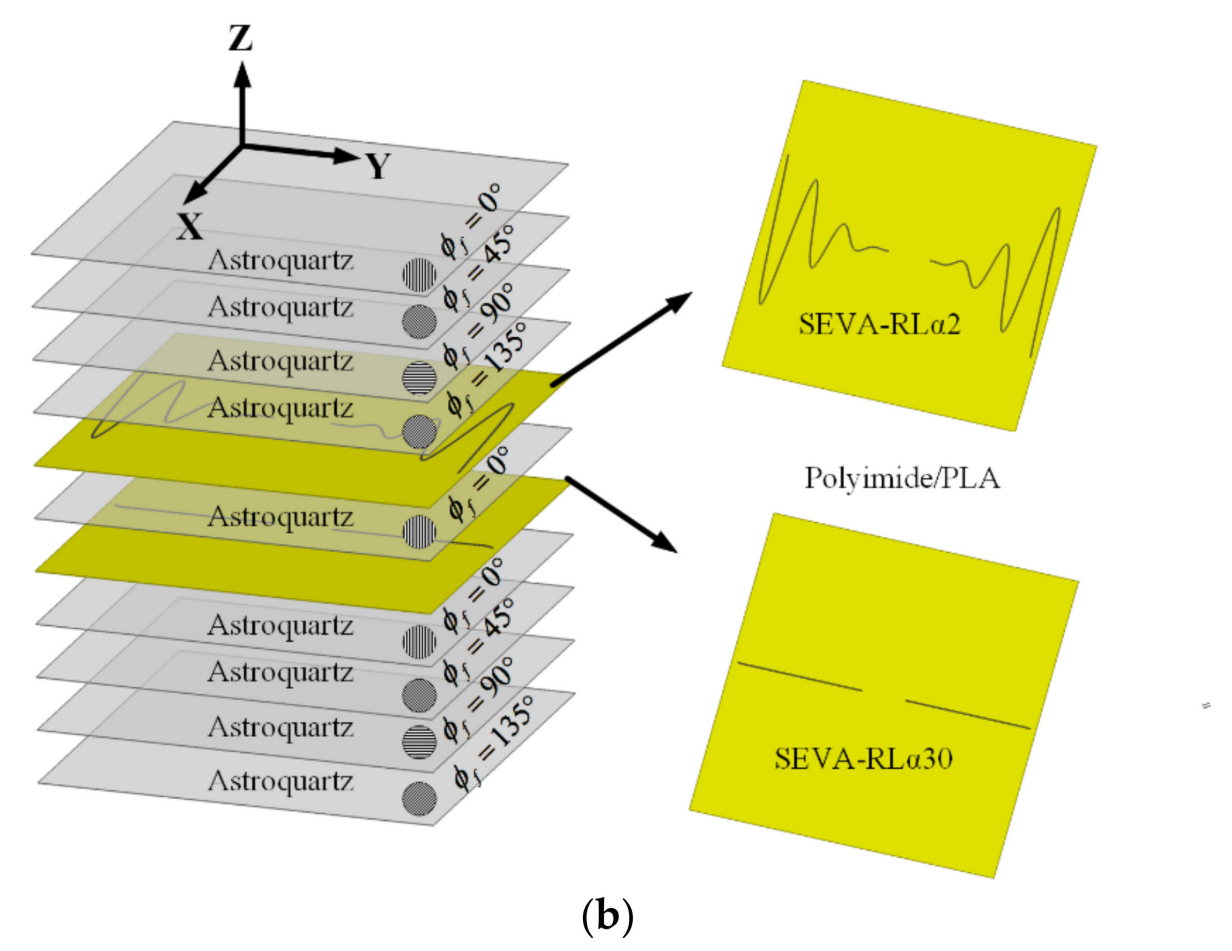
Detailed representation of multi-layer structurally embedded vascular antennas (ML-SEVA) (**a**) CAD model used for simulating the structure (**b**) Fabrication lay-up.

Figure 1 shows the CAD model and notional composite lay-up of the multi-layer SEVA (ML-SEVA) presented in this work. The design and operation of this two-layer ML-SEVA leverages advances in additive manufacturing techniques to synthesize complex co-located vascular networks that provide additional operational degrees of freedom with respect to the antenna performance metrics demonstrated by the original SEVA in [30]. The fabric lay-up for the ML-SEVA in this work features two unique vascular networks separated by a single ply low-dielectric epoxy/quartz prepreg fabric RM-2014/4581 Astroquartz^®^ III with a 0.25 mm layer thickness. Each pair of the meandered lines that form the microchannels through which liquid metal is flowed are first extrusion-printed using the sacrificial PLA formulation in a single layer thickness of 0.6 mm onto Polyimide sheets. These two pairs of meandered lines create two distinct dipole-like configurations that are then thermally transferred to a fabric layer below the previous one and arranged according to the lay-up shown in Figure 1.

The feed structure consists of parallel strips connected to the meandering microvascular elements of the antenna through antipodal dipoles; this also forms a balun-like transition [30]. Equations (1) and (2) provide the parameterization of the sacrificial PLA used to create microvascular channels for the ML-SEVA in Figure 1. These meander outward in the y-dir. starting from the feed location *y* = ±*s*_0_ = ±10.0 mm and to *y* = ±*d*_0_ = ±72.2 mm and are modulated sinusoidally in the x-dir. with a period *p*_0_ = 2 within a power series envelope with a growth parameter *α*. In this work, the first vascular network in the lay-up uses *α* = 2 to provide a sinusoidally-meandering dipole antenna topology referred to as the SEVA-RLα2. The vascular network on the layer below uses *α* = 30 to form a quasi-linear dipole-like antenna referred to as the SEVA-RLα30. The parameter *t* in these expressions denotes the location of liquid metal, or length of meandering along y-axis in SEVA, and the terms *t*_1_ and *t*_2_ (used later) denote the channel filling parameter in SEVA-RLα2 and SEVA-RLα30, respectively.

(1) $$x\left(t\right)=\frac{t^{\alpha }}{d_{0}^{\alpha -1}}\sin\left(\pi p_{0}t\right)$$

(2) $$y\left(t\right)=t+s_{0}$$

The processing steps for this structure follow [30] by first curing the composite with embedded microchannels in an autoclave. Vertical cylindrical channels are then machined down from the top of the composite to the lowest of the two sacrificial layers (SEVA-RLα30) to provide release holes for the ablation of sacrificial materials. This is followed by a post-cure in a vacuum oven to vaporize the sacrificial material and create microvascular voids in the composite. These are inspected visually and then tested with compressed air to ensure all channels cleared of any blockage. After this the vertical channel at each feed location (*x*, *y*) = (0, ±*s*_0_), with *s*_0_ = 3.94 in. (10 mm), connects the two arms of the SEVA-RLα2 and SEVA-RLα30; the remaining four only contact a single arm where they intersect *y* = ±*d*_0_ = 2.6 in.

The resulting composite with embedded microvasculature is electromagnetically functionalized by first adhering hollow copper cylindrical vias 2 mm in diameter at the two feed locations. These are extended just below the surface for mechanical stability and provide access for the pressure-driven displacement of fluids into each of the four channels. These metallic vias extend upwards through drilled holes in a 31 mil (0.7874 mm) FR4 substrate (*ε_r_* = 4.4 and *tan δ* = 0.02) where they are electrically connected (soldered) to the arms of an antipodal dipole having a length *l* = 2*y*_0_. This antipodal dipole is fed by a parallel strip feed line with a 50 Ω characteristic impedance that is terminated in a Sub-Miniature Version A (SMA) connector for measurements. In this configuration, the transition from parallel strip line to antipodal dipole operates as a Dyson-style balun for the SEVA over the frequency ranges considered in this work. The ML-SEVA operates as a combination of two thin wire dipoles growing outward from the antipodal dipole; the selective filling of channels is achieved by leaving the vertical channel at its opposing end unobstructed or sealing the individual vertical channels with a thermal adhesive where it intersects *y* = ±*d*_0_.

### 2.2. Multi-Element SEVA Conformal Linear Array (SEVA-CLA)

**Figure 2:**
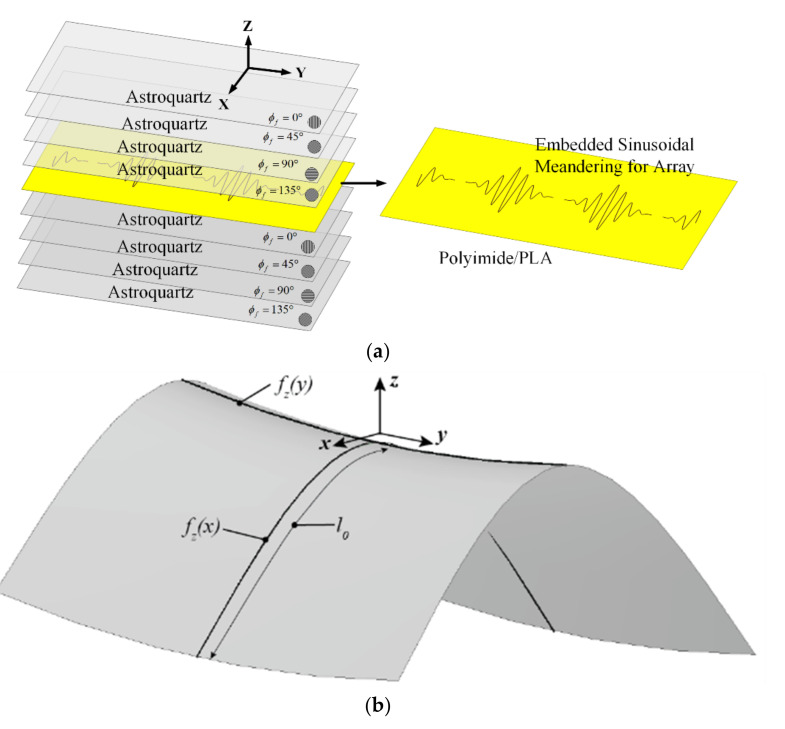
Detailed representation of multi-element SEVA conformal linear array (SEVA-CLA) (**a**) Fabrication lay-up (**b**) CAD model of complex curved mandrel used for shaping the composite.

Figure 2 shows the notional composite lay-up and CAD model of the mandrel used to form the composite into a complex-curved surface for the SEVA conformal linear array (SEVA-CLA). The complex-curved shape of the mandrel is not necessarily representative of a leading-edge design for a specific UAV and/or other aircraft, but it demonstrates the extensibility of the SEVA processing steps. This advancement in the additive manufacturing is leveraged in this work to fabricate a large contiguous channel embedded within a complex-curved composite with multiple access points. The structurally-embedded microvascular structure can operate in a number of different radiating manifold configurations; the focus here is on its operation as a three-element linear array as well as a single contiguous antenna that extends the bandwidth of the manifold. The SEVA-CLA generally follows the processing steps for the original SEVA [30] but expands on them in terms of the dimension and geometry of the complexity of the vascular network. As with other SEVA designs, debulking of the prepreg to remove volatiles from the resin and avoid the formation of porosity is an important step before curing in an autoclave. Once cured and released from the mandrel it is drilled at the prescribed locations (at the ends of the curvilinear channels) to access the sacrificial layer. The structure is then placed in a vacuum oven to post-cure and vaporize the sacrificial material and create the embedded microvascular network.

The meandering vasculature of the three-element SEVA-CLA uses the parameterization of the SEVA-RLα2 for each of the three driven antenna elements. The curvature of the mandrel used to shape the composite is approximated for the high frequency modeling of antennas by sweeping the polynomial approximations of the “leading edge” curvature $f_{z}\left(x\right)≃12.0\times 10^{-3}x^{2}$ in the xz-plane along the contour “saddle path” $f_{z}\left(y\right)\approx 275.5\times 10^{-6}y^{2}$ in the yz-plane. Three of the feed structures are first positioned then the array elements are first mapped conformally onto the complex-curved surface of the mandrel using [37]. The central element has symmetric arm lengths *d*_0_ = *d*_2_ = 3.59 in. (91.44 mm) while the arm lengths for the edge elements are asymmetric with the length of one arm fixed to *d*_0_ = *d*_1_ = 2.92 in. (74.12 mm) and the other one fixed to *d*_0_ = *d*_2_ = 3.59 in. (91.44 mm). The zero crossing points (denoted by variable *t*) on y-axis extend from 0 to 2.4 in. for sinusoidal arms of length *d*_1_ and it extends from 0 to 3.2 in. for sinusoidal arms of length *d*_2_ repeated at regular intervals of 0.4 in. along the y-axis.

**Figure 3:**
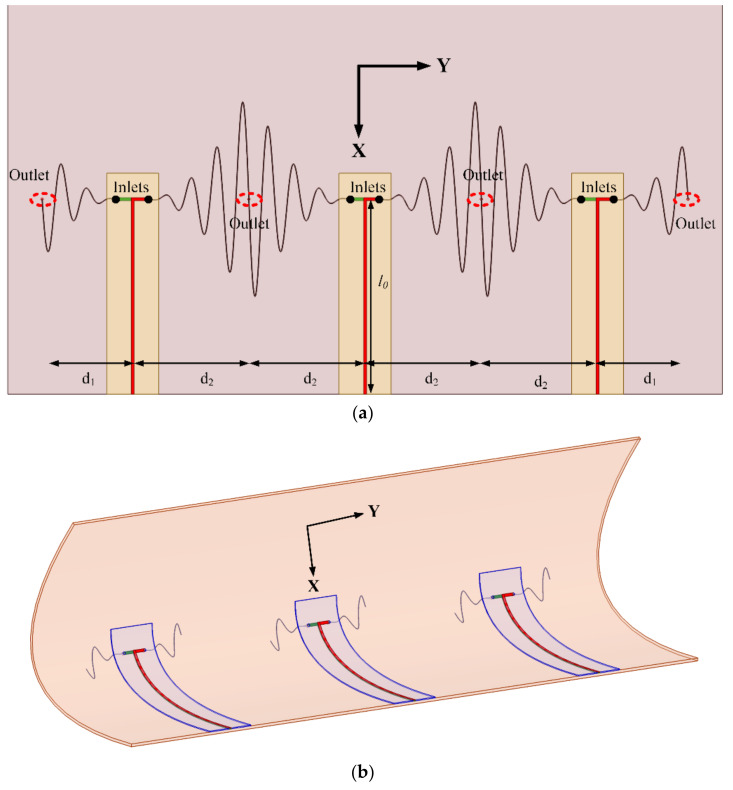
CAD Model of multi-element SEVA-CLA (**a**) Top view: Connection of feed elements with sinusoidally meandered dipoles (**b**) Side view: Connection of feed elements with sinusoidally meandered dipoles.

The topology and operation/role of the feed network for this structure is similar to the structure presented in Figure 1. Its 50 Ω impedance parallel strip feed lines have a length *l*_0_ = 5.78 in. (147 mm) and is fabricated on 31 mil (0.7874 mm) thick RT/Duroid 5880 (*ε_r_* = 2.2 and *tan δ* = 0.0004); this is used in place of the FR4 substrate to provide a feed network that can conform to the curvature of the composite. Figure 3 (top view) shows three feed elements connected to the sinusoidally meandered dipoles. Figure 3 (side view) shows the detailed connection between feed and embedded microvascular channels.

## 3. Measurement and Analysis

Discussion on the transport of liquid metal in the embedded vascular channels of ML-SEVA and SEVA_CLA is presented in this section. Comparison between simulated and measured results of these structures draw attention to the frequency reconfigurability of ML-SEVA and beam scanning ability of SEVA-CLA. Operation of SEVA-CLA as a contiguous antenna element operating in VHF is also presented in this section. Demonstration of fundamental array principles are presented by studying contour plots of isotropic antenna array and SEVA-CLA.

### 3.1. Multi-Layer SEVA (ML-SEVA)

**Figure 4:**
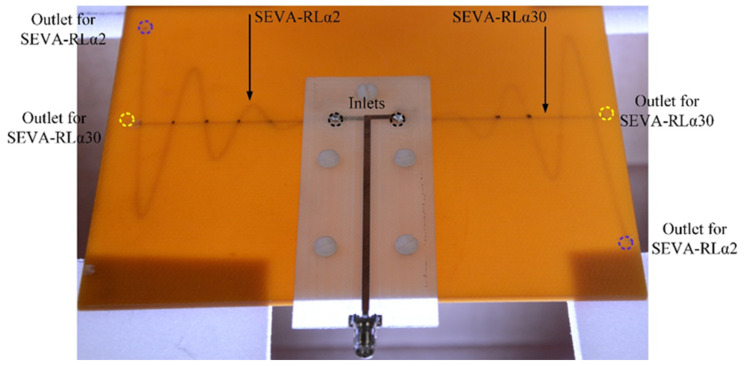
Fabricated ML-SEVA and feed network aligned and attached with nylon threads.

Figure 4 shows the final fabricated model of ML-SEVA. The embedded microvascular channels are visible in the semi-translucent ML-SEVA. This offers a means to visibility track and measure the position of liquid metal being transported through the microchannels as well as its proximity to the zero-crossing points of the exponentially increasing sinusoidal structure (SEVA-RLα2) at *t*_1_ = 0, 0.4, 0.8, 1.2, 1.6, 2, and 2.4 in. Corresponding values are chosen for *t*_2_ (SEVA-RLα30) in this experiment. Selected combinations of *t*_1_ and *t*_2_ are presented in this article to demonstrate the principle of frequency reconfiguration in ML-SEVA. Radiation patterns observed have remain consistent with simulation across different configurations and the selected combinations are shown for illustration.

Cylindrical metallic tubes with a diameter of 2 mm are soldered to the feed and visually aligned with the opening on the embedded channels in the substrate to ensure continuous and leakage-proof flow of liquid metal from inlets towards the outlets. This also establishes electrical connection between liquid metal in the embedded channels and the feed. Additionally, for mechanical stability, the feed is affixed to the substrate by nylon screws ensuring a tight fitted connection between the feed and the substrate. Filling the embedded channels with non-conductive, low-loss, low dielectric, and heat transfer fluid Fluorinert FC-70 [38] electronic liquid before flowing liquid metal ensures smooth flow of liquid metal in the channels. When liquid metal is transported from the inlet of SEVA-RLα2 towards its outlet the outlet of SEVA-RLα30 is sealed with hot melt adhesive (HMA) to avoid leakage of liquid metal into the undesired channel. Similarly, the outlet of SEVA-RLα2 is sealed with HMA when liquid metal is transported through SEVA-RLα30.

**Figure 5:**
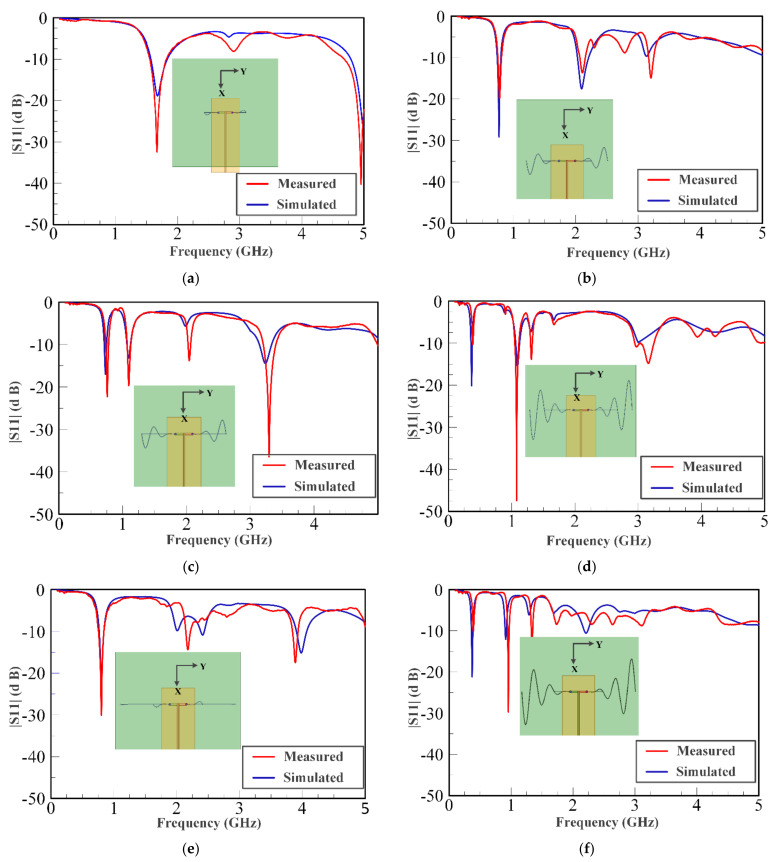

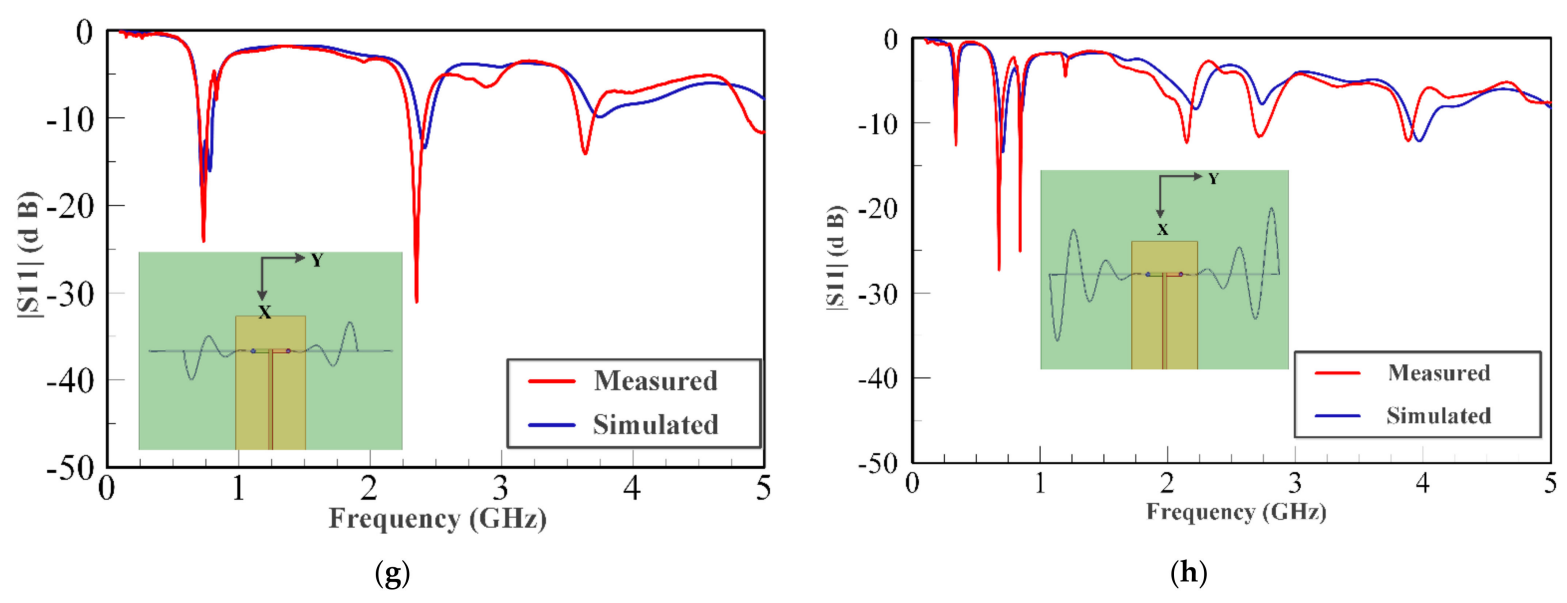
Measured and simulated S-parameter values (|S_11_| (dB)) for different scenarios. (**a**) *t*_1_ = 0.8 in. and *t*_2_ = 0.8 in. (**b**) *t*_1_ = 1.6 in. and *t*_2_ = 0.8 in. (**c**) *t*_1_ = 1.6 in. and *t*_2_ = 1.6 in. (**d**) *t*_1_ = 2.4 in. and *t*_2_ = 1.6 in. (**e**) *t*_1_ = 1.6 in. and *t*_2_ = 2.4 in. (**f**) *t*_1_ = 2.4 in. and *t*_2_ = 0.8 in. (**g**) *t*_1_ = 1.6 in. and *t*_2_ = 2.4 in. (**h**) *t*_1_ = 2.4 in. and *t*_2_ = 2.4 in.

**Figure 6:**
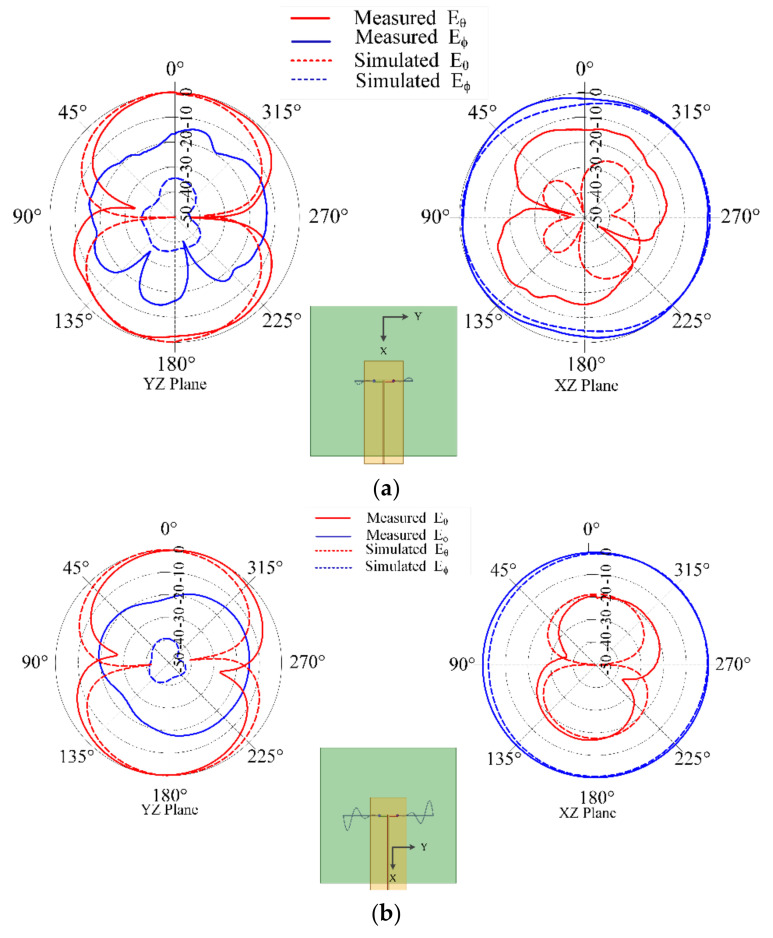

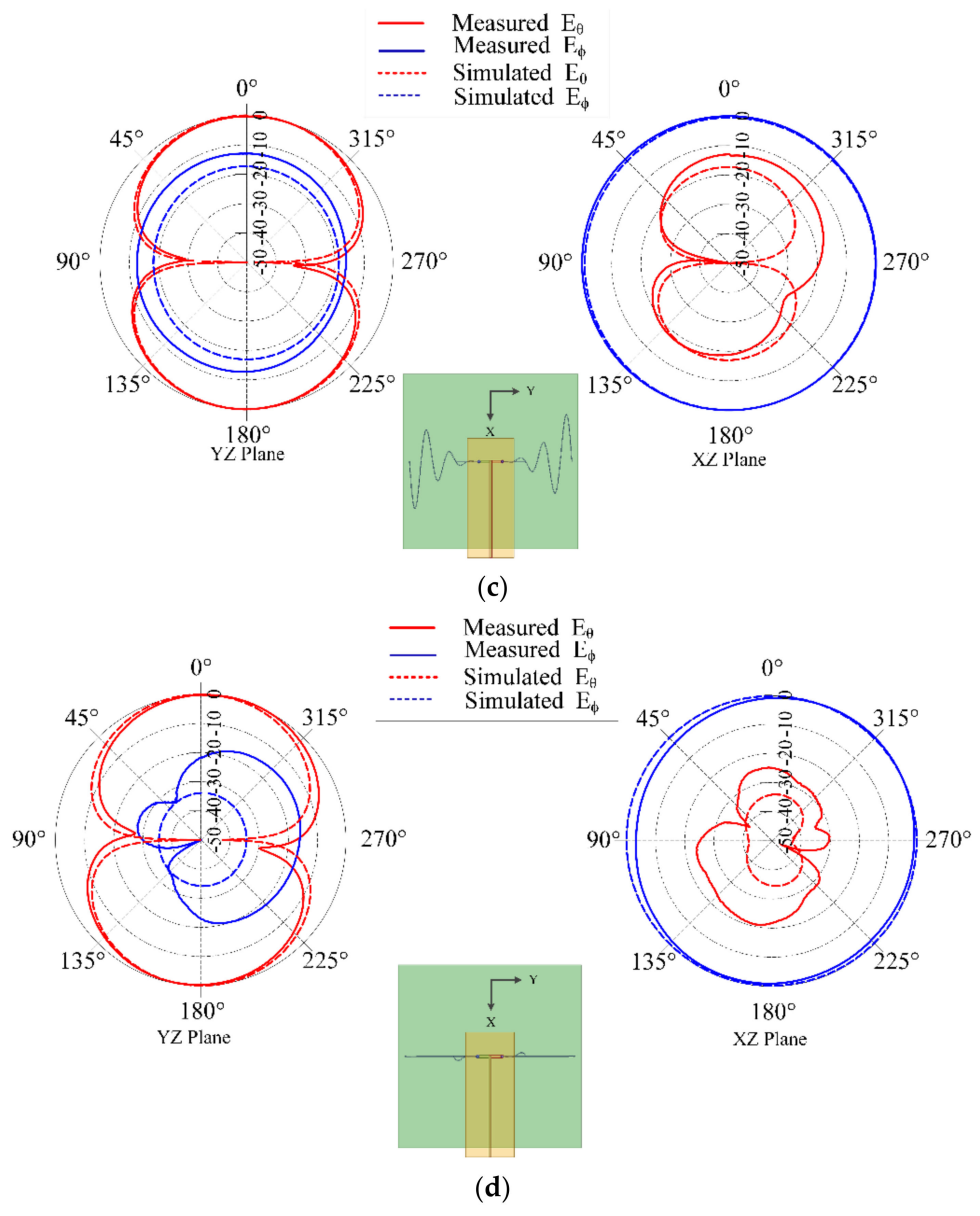
Measured and Simulated radiation pattern in yz- (left) and xz-planes (right) for different scenarios (**a**) *t*_1_ = 0.8 in. and *t*_2_ = 0.8 in. at f =1.681 GHz; (**b**) *t*_1_ = 1.6 in. and *t*_2_ = 1.6 in. at f = 0.74 GHz; (**c**) *t*_1_ = 2.4 in. and *t*_2_ = 0.8 in. at f = 0.372 GHz; and (**d**) *t*_1_ = 0.8 in. and *t*_2_ = 2.4 in. at f = 0.8 GHz.

Figure 5 shows the measured and simulated input reflection coefficient (in dB) for the chosen combinations of *t*_1_ and *t*_2_. The plots clearly demonstrate down shift of fundamental mode resonant frequency with increasing liquid metal flow in the embedded channels. These plots illustrate that the radiation pattern of the antenna remains quasi-omnidirectional for the different configurations. The close agreement observed between the measured and simulated results indicates that the operation of frequency reconfigurable ML-SEVA is also stable across the range of frequencies considered in this work. Figure 6 shows the measured and simulated *E_θ_* and *E_φ_* in xy- and xz- planes at the lowest value of magnitude (in dB) for the fundamental mode. When the antenna resonates at a frequency closer to the antipodal dipole (around 4 GHz) used to feed the microvascular component the antenna, the radiated pattern deviates from expectations due to increase interactions with the parallel strip feed network and other artifacts of experimental set-up. Thus, higher volume of metal in the embedded channels offers stable radiation pattern compared to lower metal volumes in the channels as with higher metal volumes in channel the antenna resonates at frequencies much lower than 4 GHz. Simulated maximum gains for configurations in Figure 6a–d is 2.3, −0.01, −3, 1.7, and −5 dBi, respectively.

### 3.2. Multi-Element SEVA Conformal Linear Array (SEVA-CLA)

**Figure 7:**
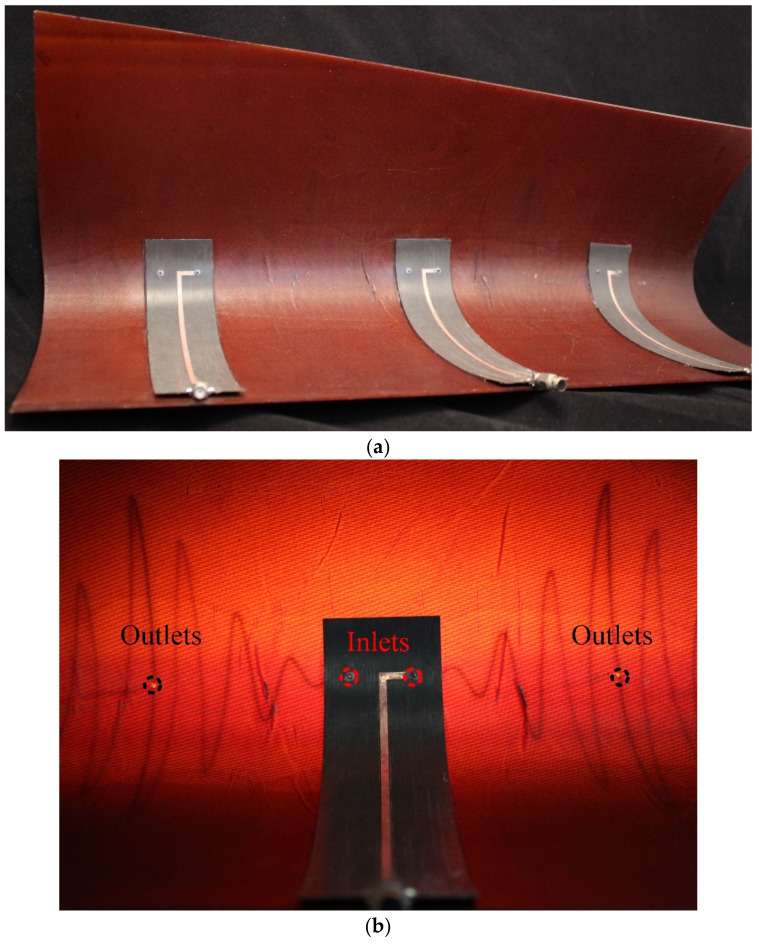
Fabricated model of SEVA-CLA (**a**) Top view showing the complete array with the three elements (**b**) Single element of the array with outlets and inlets indicated.

Figure 7 shows the fabricated model of multi-element Conformal Linear Array (SEVA-CLA). Oxidation during the fabrication of the composite resulted in an opaquer panel compared to the ML-SEVA. This limits the visibility of the embedded channels in the composite thus, the embedded channels of multi-element SEVA-CLA are filled using a different approach than ML-SEVA. Additional modifications included the use of a torus-shaped gasket formed from an HMA that was used to affix the feed structure on the curved composite. This prevents leakage of liquid metal and ensures smooth flow of liquid metal in the embedded channels.

During the measurement campaign for this antenna the embedded microchannels were first filled with FC-70 in preparation for the transport of liquid metal (to regulate back-pressure, etc.). For each of the three antennas, the antipodal dipole feed structure was first measured while the other two feeds are terminated in matched loads. Pre-measured liquid metal was then injected into the channels through the hollow via in the feed section using an appropriately tipped syringe. The amount of FC-70 flowing out from the outlets along with input reflection coefficient reading from the network analyzer was used to provide a rough estimate of the position of liquid metal in the microchannel. This was necessary as the darker color of the panel prevented visual inspection of the exact position of the fluid. This process was repeated for each element, for each of the measured configurations.

**Figure 8:**
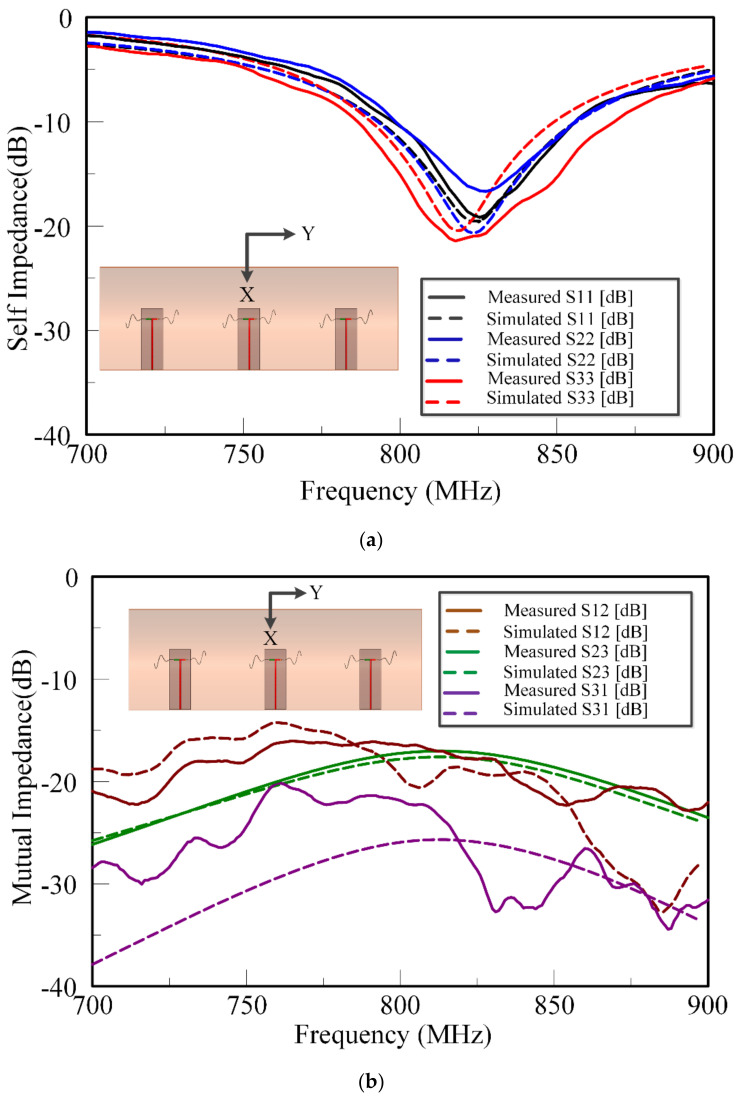
Measured and simulated impedance results of multi-element SEVACLA at 824 MHz for channel filling variable, *t* = 1.55 in (**a**) Self-impedance of the array elements (**b**) Mutual-impedance of array elements.

**Figure 9:**
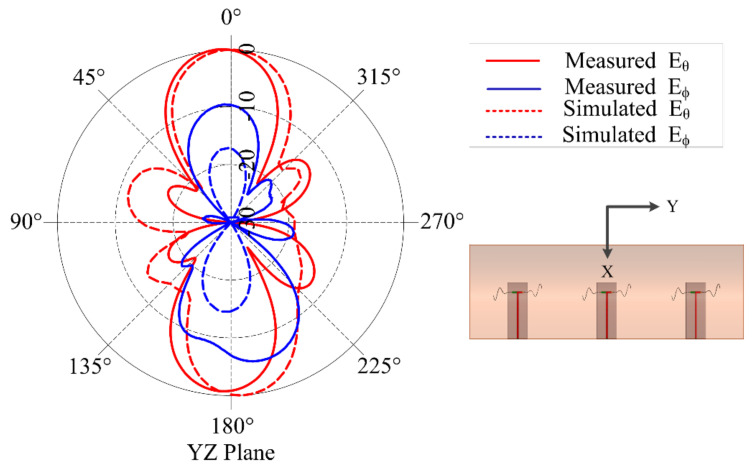
Radiation pattern of multi-element SEVA-CLA for an antenna array with 0° relative phasing at 824 MHz.

**Figure 10:**
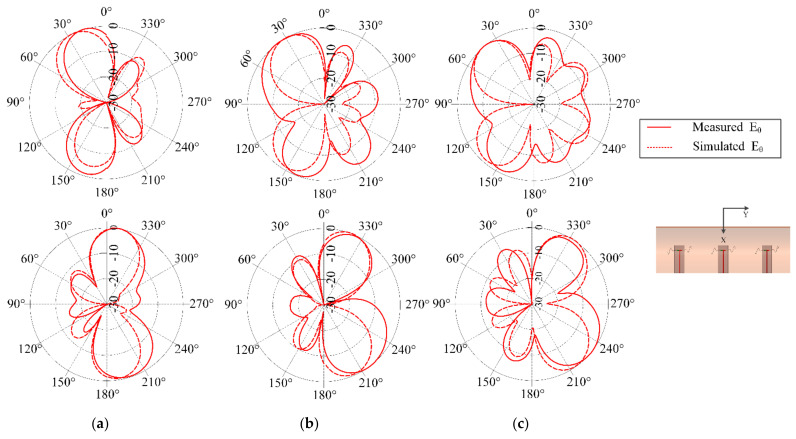
Beam scanning of multi-element SEVA-CLA for different scan angles at with channel filling factor *t* = 1.55 in. operating at 824 MHz (**a**) Radiated beam at ± 15° (**b**) Radiated beam at ± 30° (**c**) Radiated beam at ± 45°.

Figure 8 shows the simulated and measured input reflection coefficient of the multi-element SEVA-CLA with a filling of *t* = 1.55 in. in each arm of sinusoidal meandering, operating at 824 MHz as shown in Figure 3b. A subset of the mutual impedance measurements of multi-element SEVA-CLA are compared. This configuration is chosen such that the center of the matched impedance bandwidth of the antennas (when filled with liquid metal) would provide a half-wavelength element spacing in the array. Operationally, at frequencies higher than this (when the dipole arms contain less liquid metal) the spacing become much greater than a half-wavelength and the pattern behaves as expected with the generation of grading lobes and other features. The radiation pattern of SEVA-CLA with zero-degree relative phasing between the elements is shown in Figure 9. Beam scanning by the array in the yz-plane is shown in Figure 10. Maximum beam scanning range of the array is within ±45 degrees. The principal beam of the array can be scanned by varying the progressive phase shift between the elements of the array. The phase difference between the antenna elements is varied by connecting microstrip transmission lines with pre-determined phases at 824 MHz.

**Figure 11:**
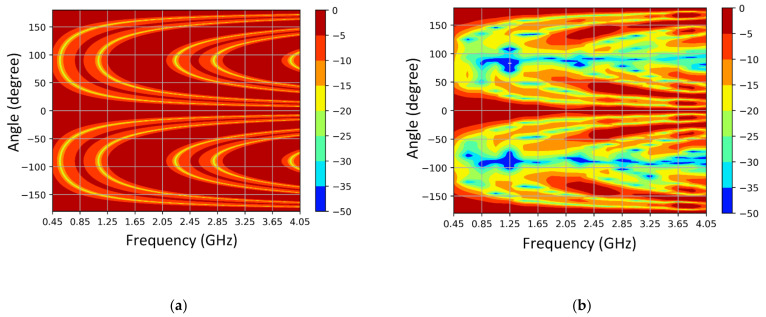
Simulated variation of radiation pattern with frequency (**a**) 3-element isotropic elements spaced 183 mm apart and (**b**) multi-element SEVA-CLA spaced 183 mm apart.

**Figure 12:**
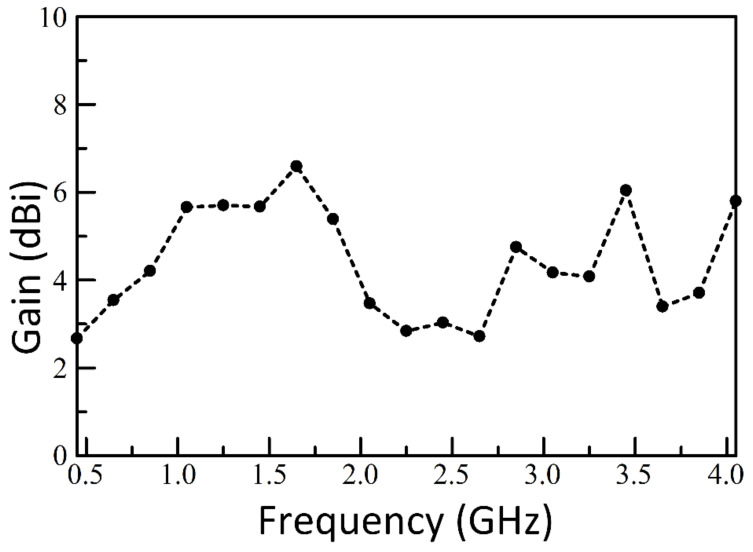
Simulated variation of gain with frequency multi-element SEVA-CLA.

The contour plot in Figure 11 shows the array pattern for three isotropic elements spaced 183 mm apart and the three element SEVA-CLA with uniform excitation over a nominal operational bandwidth from 0.45 GHz to 4.05 GHz. The overall array pattern for multi-element SEVA-CLA is obtained by multiplying the single elemental pattern of multi-element SEVA-CLA with the array pattern of similarly spaced isotropic source. Careful examination of the two normalized gain plots testifies that the gain pattern of SEVA-CLA is obtained by multiplying the gain pattern for three-element isotropic array with the unit element pattern shown in Figure 11. Additional nulls at ±90 degrees in the SEVA-CLA contour plot are introduced by the individual elemental pattern of multi-element SEVA-CLA. The position and distribution of maxima at 0 degrees and grating lobes are similar between the two plots. Increase in the number of grating lobes in the visible region at higher frequencies is due to the widening gap between individual elements in terms of wavelength. Figure 12 shows the gain distribution of SEVA-CLA over similar frequency range (0.45–4.05 GHz). The variation in the gain pattern is attributed to the coupling between the elements with different filling factor, *t*, over the frequency range. The gain of SEVA_CLA observes a dip around 2 GHz as the grating lobes start to emerge in the visible range. The efficiency of the SEVA-CLA could not be measured experimentally in the facility due to lack of available tools or in simulation but a decrease in efficiency is expected to be seen with increasing grating lobes [39].

**Figure 13:**
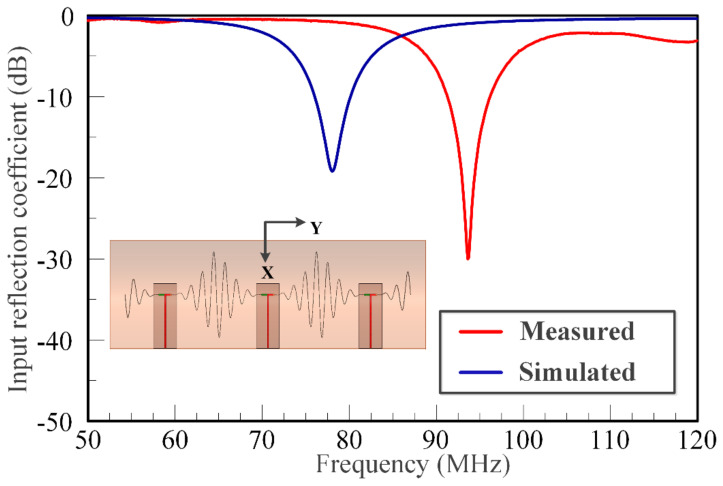
Measured and simulated results of multi-element SEVA-CLA operated as a single antenna element.

Figure 13 shows the measured input reflection coefficient for another unique configuration of the SEVA-CLA. This corresponds to the complete filling of embedded microchannels with liquid metal. The central element is driven in the configuration shown with the other two ports terminated in open circuits—this is one of many unique configurations that can potentially be achieved using different reactive loads and feed locations. In the form shown, the contiguous antenna structure operates as single antenna element with a matched impedance bandwidth centered at 95 MHz. The shift between measured and simulated input reflection coefficient is attributed to differences arising from fabrication and processing as well as other variations between modeled and fabricated designs.

## 4. Discussion

Current advances in the design and implementation of antennas engineered to reconfigure using the controlled transport of liquid metal through microchannel has been used to enable frequency reconfiguration, polarization reconfiguration, and beam-steering [40,41,42]. These designs show the wide range of applications of antenna sensors using transport of liquid metal through microchannels suitable for varied applications over wide frequency range. These and other structures are typically single-layer design with microfluidic channels traversing a planar geometry. They open up questions on the integration of these techniques for most complex host. This study demonstrates the ability to engineer multilayer and conformally curved reconfigurable antennas enabled by the transport of liquid metal in structurally embedded vascular networks. This study provides insights into the fabrication of vascular networks in two consecutive layers of fabric in the composite lay-up. It also demonstrates the ability to embed distinct patterns in a complexly curved composite. The design, fabrication and electromagnetic functionalization of these structures leads to better understanding of the scope they provide and challenges they present. These fabrication techniques have been leveraged to create multilayer SEVA in which two co-located reconfigurable antennas have been utilized synergistically for impedance and radiation tuning. In SEVA-CLA this technique has been utilized to demonstrate beam scanning of three element array on a complex curved composite which can be reconfigured for a decade of frequency. The agreement between measured and simulated data testifies the concept and ideas presented in this paper. These design techniques can be successfully utilized in designing and fabricating embedded antennas to operate as sensors in UAVs or airplanes. This research also opens avenues in designing shape-conformal electromagnetic sensors using the concepts of microfluidics and structurally embedded vascular electromagnetic devices.

## 5. Conclusions

ML-SEVA and multi-element SEVA-CLA portrays advances in design and fabrication of SEVA. Pressure driven transport of liquid metal through these structures establishes frequency-reconfiguration over a wide frequency range. Multi-element SEVA-CLA conformal to the shape of UAV wing operates as phased array establishing its beam scanning ability. These structures open avenues towards design of load bearing frequency reconfigurable antennas and antenna arrays integrable on UAVs and/or aircrafts.

## Data Availability

All the results and references are included here.

## References

[1] Inata H., Say S., Ando T., Liu J., Shimamoto S. Unmanned aerial vehicle based missing people detection system employing phased array antenna. Proceedings of the 2016 IEEE Wireless Communications and Networking Conference.

[2] Fernandez M.G., Lopez Y.A., Andres F.L.-H. (2018). On the Use of Unmanned Aerial Vehicles for Antenna and Coverage Diagnostics in Mobile Networks. IEEE Commun. Mag..

[3] Kim S.J., Lim G.J., Cho J., Côté M.J. (2017). Drone-Aided Healthcare Services for Patients with Chronic Diseases in Rural Areas. J. Intell. Robot. Syst..

[4] Tran H.H., Nguyen-Trong N., Le T.T., Park H.C. (2017). Wideband and Multipolarization Reconfigurable Crossed Bowtie Dipole Antenna. IEEE Trans. Antennas Propag..

[5] Yoon S., Tak J., Choi J., Park Y.-M. Conformal monopolar antenna for UAV applications. Proceedings of the IEEE International Symposium on Antennas and Propagation & USNC/URSI National Radio Science Meeting.

[6] Nosrati M., Jafargholi A., Pazoki R., Tavassolian N. (2018). Broadband Slotted Blade Dipole Antenna for Airborne UAV Applications. IEEE Trans. Antennas Propag..

[7] Wu D., Chen X., Yang L., Fu G., Shi X. (2017). Compact and Low-Profile Omnidirectional Circularly Polarized Antenna with Four Coupling Arcs for UAV Applications. IEEE Antennas Wirel. Propag. Lett..

[8] Hu J., Luo G.Q., Hao Z.-C. (2018). A Wideband Quad-Polarization Reconfigurable Metasurface Antenna. IEEE Access.

[9] Balderas L.I., Reyna A., Panduro M.A., Del Rio C., Gutierrez A.R. (2019). Low-Profile Conformal UWB Antenna for UAV Applications. IEEE Access.

[10] Seo D.-G., Jeong C.-H., Choi Y.-S., Park J.-S., Jeong Y.-Y., Lee W.-S. Wide Beam Coverage Dipole Antenna Array with Parasitic Elements for UAV Communication. Proceedings of the IEEE International Symposium on Antennas and Propagation and USNC-URSI Radio Science Meeting.

[11] Costa A., Goncalves R., Pinho P., Carvalho N.B. Design of UAV and ground station antennas for communications link budget improvement. Proceedings of the IEEE International Symposium on Antennas and Propagation & USNC/URSI National Radio Science Meeting.

[12] Lee C.U., Noh G., Ahn B., Yu J.-W., Lee H.L. (2019). Tilted-Beam Switched Array Antenna for UAV Mounted Radar Applications with 360° Coverage. Electronics.

[13] Zhou Y., Bayram Y., Du F., Dai L., Volakis J.L. (2010). Polymer-Carbon Nanotube Sheets for Conformal Load Bearing Antennas. IEEE Trans. Antennas Propag..

[14] Mehdipour A., Sebak A.-R., Trueman C.W., Rosca I.D., Hoa S.V. (2010). Reinforced Continuous Carbon-Fiber Composites Using Multi-Wall Carbon Nanotubes for Wideband Antenna Applications. IEEE Trans. Antennas Propag..

[15] Biswas S. Fabrication of Conformal Load Bearing Antenna using 3D Printing. Proceedings of the IEEE International Symposium on Antennas and Propagation & USNC/URSI National Radio Science Meeting.

[16] Chen C., Zheng H. Design of a dual-band conformai antenna on a cone surface for missle-borne. Proceedings of the Sixth Asia-Pacific Conference on Antennas and Propagation (APCAP).

[17] Kim J., Jang J.-Y., Ryu G.-H., Choi J.-H., Kim M.-S. (2014). Structural design and development of multiband aero-vehicle smart skin antenna. J. Intell. Mater. Syst. Struct..

[18] Xu F., Wei B., Li W., Liu J., Qiu Y., Liu W. (2015). Cylindrical conformal single-patch microstrip antennas based on three dimensional woven glass fiber/epoxy resin composites. Compos. Part B Eng..

[19] Liu Y., Du H., Liu L., Leng J. (2014). Shape memory polymers and their composites in aerospace applications: A review. Smart Mater. Struct..

[20] Bishop N.A., Miller J., Zeppettella D., Baron W., Tuss J., Ali M. (2015). A Broadband High-Gain Bi-Layer LPDA for UHF Conformal Load-Bearing Antenna Structures (CLASs) Applications. IEEE Trans. Antennas Propag..

[21] Bishop N., Ali M., Baron W., Miller J., Tuss J., Zeppettella D., Ali M. Aperture coupled MEMS reconfigurable pixel patch antenna for conformal load bearing antenna structures (CLAS). Proceedings of the 2014 IEEE Antennas and Propagation Society International Symposium (APSURSI).

[22] Kiourti A., Volakis J.L. (2014). Stretchable and Flexible E-Fiber Wire Antennas Embedded in Polymer. IEEE Antennas Wirel. Propag. Lett..

[23] Yao L., Jiang M., Zhou D., Xu F., Zhao D., Zhang W., Zhou N., Jiang Q., Qiu Y. (2011). Fabrication and characterization of microstrip array antennas integrated in the three dimensional orthogonal woven composite. Compos. Part B Eng..

[24] Zhong J., Kiourti A., Sebastian T., Bayram Y., Volakis J.L. (2016). Conformal Load-Bearing Spiral Antenna on Conductive Textile Threads. IEEE Antennas Wirel. Propag. Lett..

[25] Wang Z., Zhang L., Bayram Y., Volakis J.L. (2012). Embroidered Conductive Fibers on Polymer Composite for Conformal Antennas. IEEE Trans. Antennas Propag..

[26] Du C.-Z., Zhong S.-S., Yao L., Qiu Y.-P. Textile microstrip two-element array antenna on 3D orthogonal woven composite. Proceedings of the 2010 International Symposium on Signals, Systems and Electronics.

[27] Ghorbani K. Conformal load bearing antenna structure using Carbon Fibre Reinforced Polymer (CFRP). Proceedings of the International Workshop on Antenna Technology: Small Antennas, Novel EM Structures and Materials, and Applications (iWAT).

[28] Kim M.-S., Park C.-Y., Cho C.-M., Jun S.-M. A multi-band smart skin antenna design for flight demonstration. Proceedings of the 8th European Conference on Antennas and Propagation (EuCAP 2014).

[29] Morishita A., Kitamura C., Ohta A., Shiroma W. (2014). Two-octave tunable liquid-metal monopole antenna. Electron. Lett..

[30] Huff G.H., Pan H., Hartl D.J., Frank G.J., Bradford R.L., Baur J.W. (2017). A Physically Reconfigurable Structurally Embedded Vascular Antenna. IEEE Trans. Antennas Propag..

[31] Morales D., Stoute N.A., Yu Z., Aspnes D.E., Dickey M.D. (2016). Liquid gallium and the eutectic gallium indium (EGaIn) alloy: Dielectric functions from 1.24 to 3.1 eV by electrochemical reduction of surface oxides. Appl. Phys. Lett..

[32] Hartl D.J., Frank G.J., Baur J.W. (2016). Effects of microchannels on the mechanical performance of multifunctional composite laminates with unidirectional laminae. Compos. Struct..

[33] Hartl D.J., Frank G.J., Malak R.J., Baur J.W. (2016). A liquid metal-based structurally embedded vascular antenna: II. Multiobjective and parameterized design exploration. Smart Mater. Struct..

[34] Hartl D.J., Huff G.H., Pan H., Smith L., Bradford R.L., Frank G.J., Baur J.W. Analysis and characterization of structurally embedded vascular antennas using liquid metals. Proceedings of the Sensors and Smart Structures Technologies for Civil, Mechanical, and Aerospace Systems.

[35] Hartl D.J., Frank G.J., Huff G.H., Baur J.W. (2017). A liquid metal-based structurally embedded vascular antenna: I. Concept and Multiphysics Modelling. Smart Mater. Struct..

[36] Ansys HFSS: High Frequency Structural Simulator. https://www.ansys.com/products/electronics/ansys-hfss.

[37] Stein F.M. (1980). The Curve Parallel to a Parabola Is Not a Parabola: Parallel Curves.

[38] (2019). 3MTM FluorinertTM Electronic Liquid FC-70–3M Science Applied to Life. https://multimedia.3m.com/mws/media/64891O/fluorinert-electronic-liquid-fc-70.pdf.

[39] Vosoogh A., Kildal P.-S. (2016). Simple Formula for Aperture Efficiency Reduction Due to Grating Lobes in Planar Phased Arrays. IEEE Trans. Antennas Propag..

[40] Bharambe V.T., Adams J.J. (2020). Planar 2-D Beam Steering Antenna Using Liquid Metal Parasitics. IEEE Trans. Antennas Propag..

[41] Liu Y., Wang Q., Jia Y., Zhu P. (2020). A Frequency- and Polarization-Reconfigurable Slot Antenna Using Liquid Metal. IEEE Trans. Antennas Propag..

[42] Singh A., Goode I., Saavedra C.E. (2019). A Multistate Frequency Reconfigurable Monopole Antenna Using Fluidic Channels. IEEE Antennas Wirel. Propag. Lett..

